# Baseline Profiles of Drug Prescriptions Prior to Diagnosis of Mild Cognitive Impairment (MCI) Obtained by Latent Class Analysis (LCA), and Assessment of Their Association with Conversion to Dementia

**DOI:** 10.3390/healthcare11152219

**Published:** 2023-08-07

**Authors:** Carmen Gómez-Gómez, Miguel Ángel Moya-Molina, Manuel Jesús Tey-Aguilera, Jorge Flores-Azofra, Juan Luis González-Caballero

**Affiliations:** 1 Department of Biochemistry and Molecular Biology, School of Medicine, University of Cadiz, 11002 Cádiz, Spain; e4a.tey31@gmail.com (M.J.T.-A.); jorgifa@gmail.com (J.F.-A.); 2 Department of Neurology, Hospital Universitario Puerta del Mar (HUPM), University of Cadiz, 11009 Cádiz, Spain; 3 Department of Statistics and Operations Research, Faculty of Medicine, University of Cadiz, 11002 Cádiz, Spain; juanluis.gonzalez@uca.es

**Keywords:** mild cognitive impairment, latent class analysis, modifiable risk factors, polypharmacy, progression to dementia

## Abstract

Polypharmacy has been linked to cognitive decline. However, interventions targeting modifiable risk factors, some of which are targets of the most commonly used drugs, could reduce the prevalence of dementia. Our aim was to determine the drug prescription regimen at baseline, prior to the diagnosis of mild cognitive impairment (MCI), and its possible association with progression to dementia. Data were collected from the electronic medical records of 342 MCI outpatients diagnosed during 2006–2017 at their first neurology consultation. We followed the classical three-step method of statistical analysis, starting with a Latent Class Analysis (LCA) to discover subgroups of drug prescription probability. Half of the patients were under polypharmacy (≥5 drugs), 17.5% had no recorded medication, 33.3% progressed to dementia (94.7% in ≤5 years), and 84.1% of them to Alzheimer’s disease (AD). According to the LCA and based on 20 therapeutic indicators obtained from 240 substances and regrouped according the Anatomical Therapeutic Chemical Classification, we identified a four-profile model: (1) low (35.7% of patients); (2) mixed (28.7%); (3) cardio-metabolic (19.3%); and (4) psychotropic (16.4%). The binomial regression logistic model showed that profiles 2 and 3 (and 4 for AD), with a higher drug prescription conditioned probability against classic risk factors, were protective than profile 1 (OR = 0.421, *p* = 0.004; OR = 0.278, *p* = 0.000; OR = 0.457, *p* = 0.040, respectively), despite polypharmacy being significant in profiles 2 and 3 (mean > 7 drugs) vs. profile 1 (1.4 ± 1.6) (*p* = 0.000). Patients in the latter group were not significantly older, although being aged 65–79 years old quadrupled (OR = 4.217, *p* = 000) and being >79 tripled (OR = 2.945, *p* = 0.010) the conversion risk compared to patients <65 years old. According to the proposed analytical model, profiling the heterogeneous association of risk factors, which were taken prior to diagnosis, could be explored as an indicator of prior care and a predictor of conversion to dementia.

## 1. Introduction

Multiple chronic health conditions contribute to frailty in aging, with cognitive impairment being one of their manifestations. These conditions are often associated with polypharmacy [1,2,3,4]. Polypharmacy has been considered both a risk factor for and an indicator of cognitive status [5,6]. The potential protective or detrimental impacts on cognition of certain types of drugs has been analyzed individually for oral antidiabetics [7,8], antihypertensive and lipid-lowering agents [9], antidepressants [10], and benzodiazepines and related drugs [11]. It has been widely debated whether cognitive decline linked with polypharmacy could be attributed to the drugs used, drug–drug interactions, or the side effects of the medications [12,13]. Notably, some medications taken by older people have an anticholinergic effect (e.g., those taken for urinary frequency/incontinence, antidepressants, anti-psychotics, anti-parkinsonian, anti-arrhythmic drugs, antihistamines, bronchodilators, etc.) [14]. This has been posed as a potential explanation for the influence of a multidrug regimen on cognitive function [15].

Conversely, several studies have focused on mild cognitive impairment (MCI), a clinical entity representing a transitional state between normal cognition and dementia [16]. Not all patients with MCI necessarily deteriorate; some can remain stable or even revert to normal cognitive function over time. This depends on the diagnostic criteria, the clinical subtype [17], and the coexistence of modifiable midlife and later life risk factors, such as cardiovascular factors, cardiovascular and cerebrovascular diseases, psychiatric symptoms, and social conditions [18,19]. Moreover, early intervention targeting some of these factors has been shown to reduce the prevalence of dementia by 37% [20].

The aim of this study was to determine the baseline drug prescription regimens of MCI patients before their first neurological consultation. Given that these patients, who are typically older, are likely to be prescribed multiple drugs, Latent Class Analysis (LCA) was used to classify individuals into a limited number of multi-drug prescription subgroups. In light of the existing controversy, another objective was to assess the association between baseline medication profiles, potential polypharmacy, and the progression from MCI to a major neurocognitive disorder.

## 2. Materials and Methods

### 2.1. Data Source

This retrospective study was based on outpatients from the Department of Neurology at the Hospital Universitario Puerta del Mar (HUPM) (Cádiz, southern Spain). On arrival, neurologists examined each case according to a standardized protocol that includes clinical history, background co-morbidities, a general and neurological examination, a battery of cognitive and neuropsychiatric tests (MMSE (Mini-Mental State Examination), ADAS-cog (Alzheimer’s Disease Assessment Scale-Cognitive Subscale), ADL (Activities of Daily Living)), and blood sampling. Following this, the diagnosis of dementia is eminently clinical and based on established criteria [21,22,23,24]. MCI was diagnosed using modified Petersen criteria [25]: objective impairment in cognitive testing not severe enough to define dementia; normal general cognitive function; age- and education-corrected MMSE scored >23.8; absence of impairment in ADLs.

**Figure A1:**
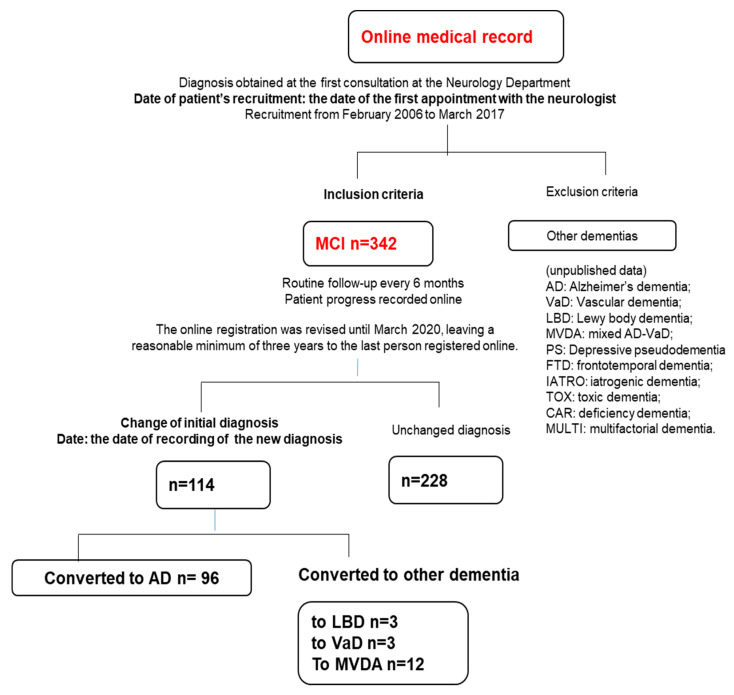
Flowchart indicating procedure followed with the patients.

This information was stored in an electronic medical record containing the patient’s history from previous examinations carried out by their General Practitioner (GP) or other specialist physicians. From this data source, all patients diagnosed with MCI for the first time between February 2006 and March 2017 were consecutively analyzed, resulting in 342 patients fulfilling the inclusion criteria (see Appendix A Figure A1). The variables were recorded in an Excel database.

### 2.2. Baseline Characteristics

Sex, age, and family history of dementia (siblings or parents) were collected. Age was categorized into three intervals: <65, 65–79, and >79 years. Currently prescribed drugs were recorded, which were taken from a datasheet provided for that purpose or from the anamnesis of other physicians. Each active compound was identified by drug term (at the 5th level), then regrouped into therapeutic group (at the 2nd level), or subgroups (at the 3rd or 4th levels) when considered more appropriate, according to the Anatomical Therapeutic Chemical Classification (ATC code) (available at www.whocc.no/atc_ddd_index/last accessed on 1 September 2021). The number of medicines prescribed was also recorded. Polypharmacy was defined as the concomitant use of ≥5 active drugs, and excessive polypharmacy as ≥10 drugs [26]. This variable was then categorized into three groups: no polypharmacy (<5 drugs); moderate polypharmacy (5–9 drugs); and excessive polypharmacy (≥10 drugs). Anticholinergic drugs scoring 3 were obtained from http://www.acbcalc.com/ (last accessed on 30 September 2021), because of their categorization as increasing the risk of dementia/impaired cognitive performance [27].

### 2.3. Conversion

As part of their regular neurological care, the patients were followed-up every 6 months. We checked medical records for possible changes in diagnosis. A “conversion interval” variable was defined as the elapsed time from the registered date of MCI diagnosis to the date of the new diagnosis. A reasonable minimum of three years after the last online registration of the individual (March 2020) was used to examine possible changes [17] (see Appendix A Figure A1).

### 2.4. Statistical Analysis

The statistical analyses were carried out irrespective of who compiled the database and the neurologist who provided the anonymized data. Categorical variables are expressed as number and percentage of the observed data; numeric variables are represented as mean ± standard deviation.

Prior to LCA, the raw data were regrouped into 20 indicators obtained by regrouping 240 substances (see above) to better reflect the main pharmacological indications and underlying medical conditions of the patients [28]. First, we explored the solution with one class to obtain the initial fitting parameters. We increased the number of classes until we obtained the best fitting model, while respecting theoretical parsimony [29]. We used several criteria, such as the Akaike Information Criterion (AIC), the Bayesian Information Criterion (BIC), the Adjusted BIC (aBIC), and the Lo–Mendell–Rubin (LMR), and bootstrap likelihood ratio (BLRT) tests. Smaller values for all information criteria (reductions of more than 10 points) were considered improvements in the model fit. An entropy value between 0 and 1 allowed us to measure the uncertainty of the classification obtained, whereby values ≥0.80 indicated a strong separation and were used identify the different classes [30,31,32,33].

Second, the distribution of variables among latent class members was analyzed. Next, the characteristic of converted vs. non-converted patients were examined and, following the classical three-step method [33], and latent class membership was included as an independent categorical variable. The association between categorical variables was contrasted using a χ2 test, or if these conditions were not verified, with Fisher’s exact test. Comparisons of quantitative variables were performed using the Student’s *t*-test.

Third, a binomial logistic regression model was performed to assess the association of baseline drug prescription profiles and other potential contributing factors to the conversion from MCI to dementia, and specifically to AD. Predictors associated with the odds ratio (OR) were estimated with a 95% confidence interval (CI) using the Wald test, with *p* < 0.05 considered statistically significant. Data were processed using IBM SPSS Statistics v24, Epidat 3.1 software, and LCA was conducted using Mplus v8.4.

## 3. Results

Of the patients, 66.5% (*n* = 225) were female and 78.1% (267) were ≥65 years old (age 72.1 ± 9.5, range = 42–97 years).

### 3.1. Drug Prescription at Baseline

The mean number of drugs used was 4.8 ± 3.8 (range = 0–20). Individuals without a prescription accounted for 17.5% (*n* = 60) of the patients, and 49.7% (*n* = 170) were under polypharmacy, 22.4% (*n* = 38) of whom were under excessive polypharmacy. Polypharmacy was significantly higher in those aged ≥65 (53.4% of these patients) compared to those <65 years old (38.7%) (*p* = 0.007).

**Figure A2:**
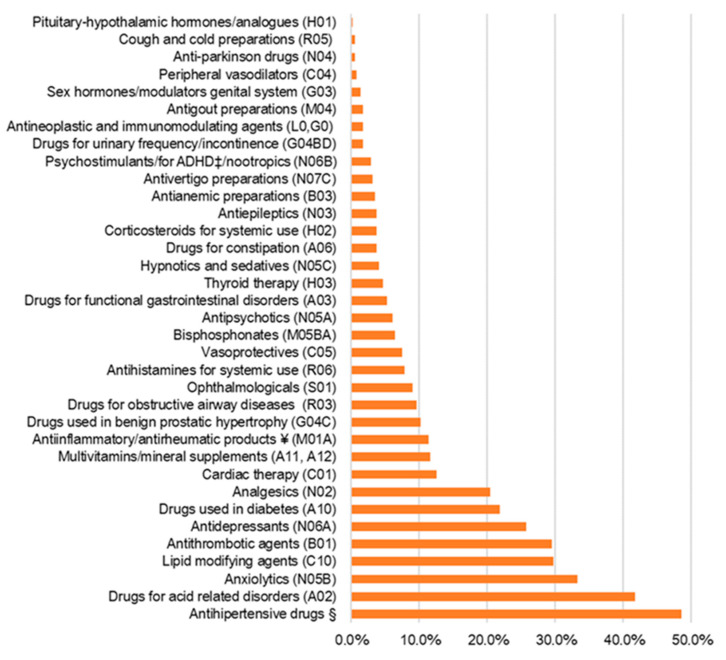
Prescription frequency of drug families at baseline. § The group “antihypertensive drugs” includes C02AC—imidazoline receptor agonists, C03—diuretics, C07—beta-blocking agents, C08—calcium channel blockers, and C089—agents acting on the renin–angiotensin system. ¥ Non-steroid products. ‡ ADHD: attention deficit hyperactivity disorder.

The most commonly used therapeutic subgroups were antihypertensive drugs (including C02AC, C03, C07, C08, and C09) (49.8% of patients, *n* = 166), drugs for acid-related disorders (A02) (42.2% *n* = 143), anxiolytics (N05B) (33%, *n* = 112), lipid-modifying agents (C10) (30.1%, *n* = 102), antithrombotic agents (B01) (29.2%, *n* = 99), antidepressants (N06A) (25.4%, *n* = 86), analgesics (N02) (20.7%, *n* = 70), and drugs used to treat diabetes (A10) (19.8%, *n* = 67) (Appendix A Figure A2).

### 3.2. LCA Model for Drug Family Prescription

The 20 indicators used for the LCA are described in detail in Appendix A (Table A1). Table 1 shows the fit criteria for each model. We observed that BIC indicated three classes, while aBIC increased this to four classes, with negligible differences with respect to the five classes (decrease of 0.826). Entropy was >0.8 in models with three or more classes. The pLMR values indicated four classes when considering the improvement of the other criteria as a whole. The adjustment for the pBRLT values was discarded because, as expected, their decline suggested more classes that would not contribute to clarification [29]. Moreover, the five-class model led to a small size profile (about 5% of the sample with 20 cases). Finally, the four-class model was chosen, allowing for a meaningful interpretation (Table 2).

According to the conditional probability shown by the indicators, we defined the class membership as follows: Class 1—patients with a small probability of having a drug prescription (labelled as low profile) (35.7% of patients, *n* = 122); Class 2—patients who had a high probability of having drug prescriptions for comorbidities from different domains (labelled as mixed profile) (28.7%, *n* = 98); Class 3—individuals who had a high probability of having prescriptions for cardio-metabolic-related medication (labelled as cardio-metabolic profile) (19.3%, *n* = 66); Class 4—patients with a high likelihood of using drugs for common psychiatric conditions (labelled as psychotropic profile) (16.4%, *n* = 56).

As a guide, the conditioned probabilities of prescribing therapeutic groups ≥0.5 are highlighted in bold in Table 2. In the low profile, a small conditional probability was found for all drugs, with the highest not exceeding 18% (antihypertensive drugs). For the mixed profile, there was no sharp break in probabilities. In this profile, it should be noted that the high probability of using analgesics (52%), followed by anxiolytic–hypnotic–sedatives (49%), antidepressants (42%), and anti-inflammatory drugs (37%), could be due more to osteo-articular rather than psychiatric problems. As cardiovascular treatments, only antihypertensive drugs were highly likely (66%). In the cardio-metabolic profile, a conditional probability ≥0.5 was observed for drugs prescribed for diabetes, antihypertensive drugs, lipid modifying agents, cardiac therapy drugs (0.45), and antithrombotic agents.

### 3.3. Inter-Class Comparison

Table 3 shows the inter-class comparisons of the patients’ demographic characteristics, family history of dementia, degree of medication, and use of high-scoring anticholinergic drugs. The sex co-variate exhibited significant differences: men were more frequently observed with a cardio-metabolic profile (53.0%), with a very high female incidence (82%) in those with a mixed profile (*p* = 0.000). Concerning the medication grade, there were significant differences (*p* = 0.000) in both the number of prescriptions and polypharmacy status. Thus, drug prescription was ≤5 in 96.7% (*n* = 118) of low profile members and in 62.5% (*n* = 35) of psychotropic profile members. Moderate polypharmacy (5–9 drugs) was present in the mixed profile (mean prescription 7.2 ± 2.9 drugs, CI95% 6.57–7.73) and in the cardio-metabolic profile (8.4 ± 3.5, CI95% 7.55–9.27), with 30.3% of class 3 members under excessive polypharmacy (≥10). The highest probability of taking prescription drugs for acid-related disorders was present in these two profiles. Significant differences (*p* = 0.021) were found in the number of drugs prescribed with anticholinergic action scored 3, the largest of which was observed in the psychotropic profile (16.1% of mem-bros).

### 3.4. Conversion

Of the sample, 33.3% of patients eventually developed dementia (*n* = 114), 84.1% (*n* = 96) of them were later re-diagnosed with AD (Alzheimer’s dementia), 2.63% (*n* = 3) with VaD (Vascular dementia), 2.63% (*n* = 3) with LBD (Lewy body dementia), and 10.53% (*n* = 12) with MVDA (mixed AD-VaD) (Table 4). The mean conversion interval was 2.55 ± 1.50 years (minimum 0.25, maximum 8.0). The cumulative annual frequency of progression was 21.1% in ≤1 year (*n* = 24); 50.9% in ≤2 years (*n* = 58), 75.4% in ≤3 years (*n* = 86); 87.7% in ≤4 years (*n* = 100); and 94.7% in ≤5 years (*n* = 108).

When comparing the converted patients vs. non-converted patients, significant differences were observed in terms of profile membership (*p* = 0.004 for all dementias, *p* = 0.003 for AD), age (*p* = 0.001, *p* = 0.005), and medication grade. Notably, for all dementias, 47.4% (*n* = 54) of the converted patients belonged to the low profile group vs. 29.8% (*n* = 68) of the non-converted; 12.3% (*n* = 14) of converted individuals were of the cardio-metabolic profile vs. 22.8% (*n* = 52) of the non-converted; 10.5% (*n* = 12) of re-diagnosed patients were aged <65 years vs. 27.6% (*n* = 63) of those who remained with MCI; 60.5% (*n* = 69) of the converted individuals were not under polypharmacy (<5 drugs) versus 45.2% (*n* = 103) of non-converted patients (*p* = 0.026). Similar data were found for AD (Table 4).

Finally, Table 5 records the results of the two binomial logistic regression analyses. The variables that did not give significant results are not shown. The low profile and <65 year groups were taken as references. The risk of progression to dementia was quadrupled in patients aged 65–79 years old (OR = 4.217, CI95% 2.092–8.500, *p* = 000) and tripled in patients aged >79 years old (OR = 2.945, CI95% 1.289–6.728, *p* = 0.010) compared to those aged <65 years. On the other hand, the mixed and cardio-metabolic profiles were protective compared to class 1 (OR = 0.421, CI95% 0.232–0.762, *p* = 0.004 and OR = 0.278, CI95% 0.137–0.565, *p* = 0.000, respectively). Regarding the conversion to AD, only the interval of 67–79 years was a risk factor for progression (OR = 3.259, CI95%1.614–6.583, *p* = 0.001), and all profiles were protective compared to class 1.

## 4. Discussion

In our study, we tried to clarify the drug prescription pattern at baseline in 342 patients diagnosed with MCI. In agreement with the literature, 33.3% progressed to AD within 5 years of onset (94.7%) [17,34]. Only 4.4% of these individuals (5.2–6.2%) developed dementia with a vascular component, also in line with the literature [17].

The four-class model from the LCA showed significant inter-cluster differences, indicating dominant morbidity patterns [35], which were summarized as musculoskeletal disorders (class 2), cardiovascular and metabolic diseases (class 3), and mental health disorders (class 4). Women were prevalent in the mixed profile (82.7% membership) and men were prevalent in the cardio-metabolic profile (53%) (*p* = 0.000), in line with studies in similar populations [36,37]. Regarding polypharmacy, a higher prevalence was observed compared to a study based on a Spanish primary care database: polypharmacy (≥5) was observed in 49.7% of our sample vs. 23.4% of the 65–79-year-old group and 36.7% of those aged ≥80 years; excessive polypharmacy (≥10) was observed in 11.1% of the sampled individuals vs. 2.61% of the 65–79-year-old group and 4.78% of those aged ≥80 years [38]. Thus, we cannot rule out that polypharmacy may have played a role in the cognitive frailty of our patients, 78.1% of whom were aged ≥65 years (mean 72.1 ± 9.5 years) (approximately 15–20% of people ≥65 years could have MCI) [39]).

Surprisingly, 47.4% of patients converted to dementia vs. 29.8% who did not convert (*p* = 0.004) (50.0% vs. 30.1% for AD, *p* = 0.003), which belonged to the low prescription profile. Additionally, the binomial regression analysis showed the other profiles to be protective against conversion. Moreover, patients with mixed and cardio-metabolic profiles were significantly (*p* = 0.000) subjected to polypharmacy (7.15 ± 2.89 and 8.41 ± 3.49 drugs prescribed, respectively) vs. the low profile group (1.35 ± 1.63). This was not a question of age, as it should be noted that the age difference between the profiles was at the limit of statistical significance (*p* = 0.05). It is true that, in the binomial regression model, age was the other risk factor for progression, which is to be expected: with <65 years as the reference group, there was a four-fold increase in risk at 65–79 years (for all dementias or AD) and a three-fold increase in risk at >80 years (for overall dementia).

Could the combination of pharmacological treatments targeting the underlying pathologies that are considered modifiable risk factors in middle and old age [20], which are also mutually reinforcing [40,41,42], was effective? In our “protective profiles”, the most frequently prescribed drugs were directed against hypertension, altered blood lipids, vascular protection, diabetes, pain (with its implications for physical inactivity and psychological disturbance) [43], depression, anxiety, and insomnia [44]. Most studies point to the association of polypharmacy or inappropriate medication with cognitive decline [11,45,46,47], even with daily consumption of ≥3 drugs [13]. The worsening of cognitive function attributable to antidepressants or benzodiazepines [48] argues against our finding of a protective role for the psychotropic profile. However, other authors reported that pharmacological intervention, even targeting cardiovascular risk factors, has no effect on the cognitive status [49]. For a similar home-dwelling population (74.7 ± 3.9 years, 63.9% females), no differences were found in medication grade between remaining or converted MCI, with the mean intake being comparable in both cases (4.08 ± 2.52 and 4.8 ± 3.8, respectively) to our results (4.31 ± 3.03) [50].

Nevertheless, a comprehensive study on the role of “protectan” combinations (vitamins, minerals, herbals) in preventing/delaying dementia, as opposed to complex drug associations, found that polypharmacy putatively facilitated cognitive decline while potentially favoring exposure to "protectans" [51]. They even found that the reversal of cognitive decline attributable to the Metabolic Enhancement for Neurodegeneration protocol (MEND) could be due to cardio-metabolic and anti-inflammatory medications, combined with the use of supplements and a healthy lifestyle. The methodological heterogeneity used to establish medication–cognitive impairment associations probably makes it difficult to contrast polypharmacy studies, contributing to the controversy.

As for the underlying comorbidities, one study noted that progression to AD was not influenced by the above-mentioned risk factors [52]. Therefore, let us assume that the converted patients could be in an early stage of AD before MCI diagnosis, and hence, the low profile membership (low comorbidity). However, it has been shown that about 80% of autopsied AD brains show compatible changes with other causes of dementia [18] and that, after decades-long latency periods from the initial pathological changes, the onset of symptoms is possibly triggered by environmental factors [53,54]. In this matter, recent LCA studies have shown that low morbidity profiles (7.5% diabetes, 8% depression, 25% hypertension, or 7% physical inactivity) exhibited a higher rate of MCI reversal [55].

Lastly, it has been emphasized that high standards of usual care [56] and appropriate treatment (especially of hypertension and diabetes) can prevent/delay the progression of MCI to dementia. Patients “hidden in the community”, without family and medical support, have a higher severity and prevalence of MCI [57]. Given the characteristics of the Spanish public healthcare system, most of our patients were referred to the neurologist from primary care services. The general population has regular access to these services and they are followed-up within prevention programs, although this entails polypharmacy. Perhaps our results reflect not so much the successful effect of prior medication, but of care, on the potential progression of MCI. Low drug prescription levels at baseline may reflect that these individuals have not seen their GP, by carelessness or because they did not need it. When they finally did, it was the symptoms were severe, because they, or their family, already perceived the cognitive impairment as suspicious of something more serious.

### Limitations

Our study had some limitations, including the small sample size, the unavailability of data on the patients’ adherence to treatments, the lack of the starting date of each treatment, the unavailability of reversed cases, not including how long patients had been in MCI conditions prior to receiving the most accurate neurological diagnosis, and the unavailability of lifestyle parameters in the medical records. As we used written sources and not patient interviews, comorbidities were defined when patients had an altered parameter or were on treatment (e.g., hypertension, dyslipidemia, or diabetes). Thus, the same variable could have been repeated twice. Therefore, although they were collected (unpublished data), they were not included in this study.

## 5. Conclusions

At baseline, prior to the diagnosis of MCI, four drug prescription profiles were detected, indicating dominant morbidity patterns. In these patients who progressed to dementia according to the rates, intervals, and types reported in the literature, the profiles with a higher conditional probability of prescription (mixed, cardio-metabolic, and psychotropic) were shown to be protective against conversion to dementia, compared to the low profile. Their members were not significantly older, although age was certainly a risk factor. Beyond drug consumption, they could somewhat reflect previous patient care.

The classical three-step statistical analysis could be a tool for finding subjacent profiles in patients subjected to complex associations of variables. Based on baseline data collected from electronic medical records, this profiling could be explored at baseline as a predictor of the risk of dementia progression in MCI patients at onset.

## Figures and Tables

**Table 1:** Fit parameters in latent class analysis.

Model	Nparameters	LL (df)	AIC	BIC	aBIC	Entropy	*p*VLMR	*p*BLRT
1 cl	20	−2964.356	5968.711	6045.407	5981.963	na	na	na
2 cl	41	−2782.864	5647.728	5804.955	5674.894	0.787	0	0
3 cl	62	−2712.092	5548.184	5785.942	5589.265	0.826	0.4118	0
4 cl	83	−2668.776	5503.552	5821.842	5558.547	0.814	0.027	0
5 cl	104	−2640.406	5488.812	5887.632	5557.721	0.882	0.2653	0.004
6 cl	125	−2616.67	5483.741	5963.092	5566.564	0.851		0.144

**Table 2:** Conditional probability of prescription according to drug family.

	No. Individuals (%)	Class 1 (Low Profile)	Class 2 (Mixed Profile)	Class 3 (Cardio-Metabolic Profile)	Class 4 (Psychological Profile)
Class prevalence (%)		35.7	28.7	19.3	16.4
No. individuals		122	98	66	56
Indicator		Conditioned probability			
Drugs used to treat diabetes	75 (21.9)	0.129	0.183	**0.525**	0.113
Drugs used as antihypertensives	167 (48.8)	0.181	**0.659**	**0.918**	0.304
Lipid-modifying agents	102 (29.8)	0.094	0.229	**0.767**	0.291
Cardiac therapy	43 (12.6)	0	0.078	0.445	0.095
Antithrombotic agents	101 (29.5)	0.095	0.162	**0.928**	0.205
Vasoprotectives/peripheral vasodilators	28 (8.2)	0.027	0.099	0.124	0.113
Antidepressants	88 (25.7)	0.037	0.418	0.088	**0.606**
Anxiolytic–hypnotic–sedatives	122 (35.7)	0	**0.49**	0.286	**0.913**
Antipsychotics	21 (6.1)	0.015	0.059	0.036	0.188
Drugs for other neurological diseases	25 (7.3)	0.035	0.039	0.09	0.191
Pain treatment	79 (23.1)	0.05	**0.52**	0.237	0.071
Anti-inflammatory products	51 (14.9)	0.033	0.366	0.118	0.032
Bisphosphonates	22 (6.5)	0.016	0.181	0.024	0
Drugs for acid-related disorders	143 (41.8)	0.03	**0.74**	**0.818**	0.16
Drugs for other digestive disorders	30 (8.8)	0	0.228	0.077	0.025
Drugs for urinary tract	40 (11.7)	0.044	0.102	0.295	0.085
Drugs for COPD-asthma	53 (15.5)	0.035	0.293	0.225	0.069
Ophthalmologicals	31 (9.1)	0.081	0.127	0.108	0.026
Drugs for deficiencies	46 (13.5)	0.039	0.288	0.132	0.055
Thyroid therapy	16 (4.7)	0.021	0.079	0.029	0.062

Drug prescription probability ≥0.5 is highlighted in bold to facilitate interpretation.

**Table 3:** Comparison of individual characteristics between classes.

	TOTAL	Class 1	Class 2	Class 3	Class 4	*p* *
No. individuals	342	122	98	66	56	
Sex (female), *n* (%)	225 (66.5)	78 (63.9)	81 (82.7)	31 (47.0)	35 (63.6)	0.000
Age interval, *n* (%)						
<65 years	75 (21.9)	30 (24.6)	21 (21.4)	6 (9.1)	18 (32.1)	0.05
65–79 years	200 (58.5)	69 (56.6)	61 (62.2)	45 (68.2)	25 (44.6)	
>79 years	67 (19.6)	23 (18.8)	16 (16.3)	15 (22.7)	13 (23.2)	
Family history of dementia, *n* (%)	51 (14.9)	18 (14.8)	12 (12.2)	11 (16.7)	10 (17.9)	0.775
Medication grade						
No. of drugs (means ± SD)	4.8 ± 3.8	1.4 ± 1.6	7.2 ± 2.9	8.4 ± 3.5	4.2 ± 1.8	0.000
CI95	4.43–5.25	1.06–1.64	6.57–7.73	7.55–9.27	3.69–4.67	
Polypharmacy status (*n*,%)						
Non-polypharmacy, <5	172 (50.3)	118 (96.7)	14 (14.3)	5 (7.6)	35 (62.5)	0.000
Moderate polypharmacy, 5–9	132 (38.6)	4 (3.3)	66 (67.3)	41 (62.1)	21 (37.5)	
Excessive polypharmacy, ≥10	38 (11.1)	0 (0.0)	18 (18.4)	20 (30.3)	0 (0.0)	
Anticholinergic score 3, *n* (%) ^¥^	28 (8.2)	5 (4.1)	11 (11.2)	3 (4.5)	9 (16.1)	0.021

* χ2 test; ^¥^ The prescribed drugs with an anticholinergic score of 3 (http://www.acbcalc.com accessed on 30 September 2021) were hydroxyzine, olanzapine, amitriptyline, paroxetine, solifenacin, tolterodine, trihexiphenidyl, and clomipramine.

**Table 4:** Characteristics of patients progressing to dementia.

	CONV	NCONV	*p* *	CONV AD †	NCONV AD	*p* *
TOTAL, *n* (%)	114 (33.3)	228 (66.7)		96 (28.1)	246 (79.9)	
Class, *n*% §						
1 (low profile)	54 (47.4)	68 (29.8)	0.004	48 (50.0)	74 (30.1)	0.003
2 (mixed profile)	26 (22.8)	72 (31.6)		25 (26.0)	73 (29.7)	
3 (cardio-metabolic profile)	14 (12.3)	52 (22.8)		10 (10.4)	56 (22.8)	
4 (psychotropic profile)	20 (17.5)	36 (15.8)		13 (13.5)	43 (17.5)	
Sex (female), *n* (%)	80 (70.2)	145 (63.6)	0.248	68 (70.8)	157 (63.8)	0.242
Age interval, *n* (%)						
<65	12 (10.5)	63 (27.6)	0.001	12 (12.5)	63 (25.7)	0.005
65–79	79 (69.3)	121 (53.1)		69 (71.9)	131 (53.5)	
>79	23 (20.2)	44 (19.3)		15 (15.6)	52 (20.8)	
Family history of dementia, *n* (%)	15 (13.2)	36 (15.8)	0.520	14 (14.6)	37 (15.4)	0.915
Medication grade						
No. of drugs (means ± SD)	4.1 ± 3.7	5.2 ± 3.8	0.014	4.0 ± 3.8	5.2 ± 3.8	0.014
CI95	3.44–4.80	4.69–5.70		3.27–4.80	4.68–5.63	
Polypharmacy status, *n* (%)						
<5 drugs	69 (60.5)	103 (45.2)	0.026	59 (61.5)	113 (45.9)	0.004
5–9 drugs	36 (31.6)	96 (42.1)		29 (30.2)	103 (41.9)	
≥10 drugs	9 (7.9)	29 (12.7)		8 (8.3)	30 (12.2)	
Anticholinergic score 3, *n* (%)	10 (8.8)	18 (7.9)	0.780	7 (7.3)	21 (8.5)	0.706

* χ2 test; § cluster membership is considered an independent variable; CONV: patients converted to dementia; NCONV: patients not converted to dementia; CONV AD: † patients converted to AD (without vascular component); NCONV AD: remaining individuals.

**Table 5:** Binomial regression logistic model to assess the risk of MCI conversion to dementia.

	Coef.	SE	Wald	g	*p*	OR	CI95%
ALL DEMENTIAS							
<65 years old referent						1	
65–79 years old	1.439	0.358	16.197	1	0.000	4.217	2.092–8.500
>79 years old	1.080	0.421	6.570	1	0.010	2.945	1.289–6.728
class 1 referent			15.993	3	0.001		
Class 2	−0.866	0.303	8.176	1	0.004	0.421	0.232–0.762
Class 3	−1.281	0.362	12.530	1	0.000	0.278	0.137–0.565
Class 4	−0.295	0.351	0.707	1	0.400	0.744	0.374–1.481
Constant	−1.304	0.343	14.437	1	0.000	0.271	
AD							
<65 years old referent						1	
65–79 years old	1.182	0.359	10.852	1	0.001	3.259	1.614–6.583
>79 years old	0.504	0.446	1.282	1	0.258	1.656	0.692–3.966
class 1 referent			16.274	3	0.001		
Class 2	−0.733	0.305	5.779	1	0.016	0.480	0.264–0.873
Class 3	−1.480	0.399	13.756	1	0.000	0.228	0.104–0.498
Class 4	−0.783	0.384	4.150	1	0.042	0.457	0.215–0.971
Constant	−1.216	0.342	12.657	1	0.000	0.296	

## Data Availability

Data are not publicly available. Correspondence and requests for materials should be addressed to C.G.-G.

## Appendix A

**Table A1:** Description of indicators used in LCA that resulted from drug family regrouping.

1. Drugs used to treat diabetes (A10) ^§^	
blood-glucose-lowering drugs (A10B)	insulins and analogues (A10A)
2. Lipid-modifying agents (C10A)	
HMG CoA reductase inhibitors (C10AA) fibrates (C10AB)	other lipid-modifying agents (C10AX09)
3. Drugs used as antihypertensives	
Antiadrenergic agent (C02AC) diuretics (C03) beta blocking agents (C07)	calcium channel blockers (C08) agents acting on the renin-angiotensin system (C09)
4. Cardiac therapy (C01)	
cardiac glycosides (C01A) antiarrhythmic, class I and III (C01B)	vasodilators (C01D), alone or combined other cardiac preparations (C01E)
5. Antithrombotic agents (B01A)	
vitamin K antagonist (B01AA) heparin group (B01AB)	direct thrombin inhibitor (B01AE) platelet aggregation inhibitors (B01AC)
6. Vasoprotectives (C04)/peripheral vasodilators (C05)	

7. Antidepressants (N06A)	
SSRI ^†^ (N06AB) other mechanism (N06AX)	non-selective monoamine reuptake inhibitors (N06AA)
8. Anxiolytic–hypnotic–sedatives	
benzodiazepines: anxiolytic (N05BA); hypnotic–sedatives (N05CD) other hypnotic–sedatives, no barbiturates (N05CF, N05CM)	
9. Antipsychotics (N05A)	
from all groups (N05AA to N05AX)	
10. Drugs for other neurological uses	
anti-Parkinson drugs (N04) anti-epileptics (N03)	antivertigo preparations (N07C) psychostimulants, drugs to treat ADHD, nootropics (N06B)
11. Drugs for paint treatment	
analgesic opioid (N02A) and other analgesic–antipyretics (N02B) other drugs used (N03, A03, DO4AB)	
12. Anti-inflammatory products	
anti-inflammatory/antirheumatic products, non-steroidal (M01A) (M02A) glucocorticoids for systemic use (H02AB)	
13. Osteoporosis (bisphosphonates [M05BA])	

14. Drugs for acid related disorders (A02)	
antacids (A02A)	proton pump inhibitors, H_2_-receptor antagonists (A02B)
15. Drugs for other digestive disorders	
drugs for functional gastrointestinal disorders (A03) drugs for constipation (A06)	
16. Drugs for urinary tract	
benign prostatic hypertrophy (G04C)	urinary frequency/incontinence (G04BD)
17. Drugs for COPD-asthma	
obstructive airway diseases (R03) cough and cold preparation (R05)	antihistamines for systemic use (R06)
18. Ophthalmologicals (S01)	

19. Drugs for deficiencies	
vitamins and minerals (A11, 12)	antianemic preparations (B12, folic acid, iron) (B03)
20. Thyroid therapy (H03)	

^§^ in parentheses is the ACT nomenclature of the drug subgroup including in each item; ^†^ SSRI: selective serotonin reuptake inhibitor.
